# Ca^2+^‐activated Cl^−^ channels (TMEM16A) underlie spontaneous electrical activity in isolated mouse corpus cavernosum smooth muscle cells

**DOI:** 10.14814/phy2.15504

**Published:** 2022-11-16

**Authors:** Xin Rui Lim, Bernard T. Drumm, Gerard P. Sergeant, Mark A. Hollywood, Keith D. Thornbury

**Affiliations:** ^1^ Smooth Muscle Research Centre Dundalk Institute of Technology Dublin Ireland

## Abstract

Penile detumescence is maintained by tonic contraction of corpus cavernosum smooth muscle cells (CCSMC), but the underlying mechanisms have not been fully elucidated. The purpose of this study was to characterize the mechanisms underlying activation of TMEM16A Ca^2+^‐activated Cl^−^ channels in freshly isolated murine CCSMC. Male C57BL/6 mice aged 10–18 weeks were euthanized via intraperitoneal injection of sodium pentobarbital (100 mg.kg^−1^). Whole‐cell patch clamp, pharmacological, and immunocytochemical experiments were performed on isolated CCSM. Tension measurements were performed in whole tissue. TMEM16A expression in murine corpus cavernosum was confirmed using immunocytochemistry. Isolated CCSMC developed spontaneous transient inward currents (STICs) under voltage clamp and spontaneous transient depolarizations (STDs) in current clamp mode of the whole cell, perforated patch clamp technique. STICs reversed close to the predicted Cl^−^ equilibrium potential and both STICs and STDs were blocked by the TMEM16A channel blockers, Ani9 and CaCC(inh)‐A01. These events were also blocked by GSK7975A (ORAI inhibitor), cyclopiazonic acid (CPA, sarcoplasmic reticulum [SR] Ca^2+‐^ATPase blocker), tetracaine (RyR blocker), and 2APB (IP_3_R blocker), suggesting that they were dependent on Ca^2+^ release from intracellular Ca^2+^ stores. Nifedipine (L‐type Ca^2+^ channel blocker) did not affect STICs, but reduced the duration of STDs. Phenylephrine induced transient depolarizations and transient inward currents which were blocked by Ani9. Similarly, phenylephrine induced phasic contractions of intact corpus cavernosum muscle strips and these events were also inhibited by Ani9. This study suggests that contraction of CCSM is regulated by activation of TMEM16A channels and therefore inhibition of these channels could lead to penile erection.

## Graphical abstract

This study suggests a simple model of mechanism of TMEM16A channels activation: Ca^2+^ influx via CRAC channel; and spontaneous Ca^2+^ release from the SR, initiated via RyR and possibly propagated via IP_3_R. Released Ca^2+^ then activates TMEM16A channels to depolarise membrane potential, thus activating L‐type Ca^2+^ channels to cause further depolarisation. Adrenergic receptor activation can cause activation of TMEM16A channels through the IP_3_R pathway.

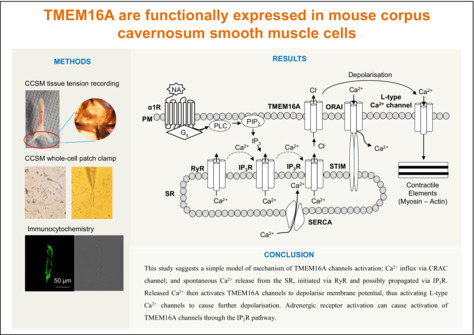

## 1 INTRODUCTION

Penile erection occurs when corpus cavernosum smooth muscle (CCSM) relaxes, allowing the expansion of corporal sinusoids as they are filled with blood. This creates turgidity within the surrounding fibrous capsule, the tunica albuginea (Andersson & Wagner, 1995). Conversely, contraction of the CCSM minimizes blood flow to limit erection, resulting in penile flaccidity (Andersson & Wagner, 1995). Contraction of CCSM is ultimately determined by the intracellular Ca^2+^ concentration, which facilitates the actin‐myosin complex. Ca^2+^ release from intracellular stores, and/or the influx of extracellular Ca^2+^ can contribute to the rise in intracellular Ca^2+^. Importantly, L‐type Ca^2+^ channel blockers reduce the amplitude of both spontaneous and agonist‐induced contractions of CCSM, suggesting that depolarization of membrane potential plays an important role in maintaining detumescent tone (Hashitani et al., 2005; Höppner et al., 1996; McCloskey et al., 2009). However, the cellular mechanisms that regulate membrane potential in CCSMC are still unclear.

So far, a variety of ion channels including large conductance calcium‐activated potassium channels (BK_Ca_) channels (Hannigan et al., 2016; Lee & Kang, 2001; Werner et al., 2005), K_v_ channels (Malysz et al., 2001; Malysz et al., 2002), L‐and T‐type voltage‐gated Ca^2+^ channels (Höppner et al., 1996; McCloskey et al., 2009) and voltage‐gated Na^+^ (Na_V_) channels (Lim et al., 2022) have been found in CCSM myocytes (for review see Thornbury et al., 2019). There is also evidence that Ca^2+^‐activated Cl^−^ channels are expressed (Craven et al., 2004; Hannigan et al., 2017; Karkanis et al., 2003; Williams & Sims, 2007). In 2008 the molecular identity of Ca^2+^‐activated Cl^−^ channels was shown to be TMEM16A (Caputo et al., 2008; Schroeder et al., 2008; Yang et al., 2008) and transcripts for *ANO1*, the gene encoding TMEM16A, have been detected in rabbit CCSM (Hannigan et al., 2017). The resting membrane potential (RMP) of rabbit CCSM lies within the range − 50 mV to −41 mV (Hashitani et al., 2005), while the Cl^−^ equilibrium potential (E_Cl_) in various smooth muscle types falls in the range − 38 mV to −19 mV (Chipperfield & Harper, 2000). Thus, activation of Ca^2+^‐activated Cl^−^ channels in CCSM cells at the RMP would be expected to result in an efflux of Cl^−^ ions, generating an inward current, membrane depolarization and resultant opening L‐type Ca^2+^ channels (Large & Wang, 1996).

In the present study, we examined the mechanisms underlying spontaneous Ca^2+^‐activated Cl^−^ currents in murine CCSMC. It is important to characterize this activity in murine tissues as murine models are commonly used to study erectile dysfunction (Hedlund et al., 2000; Werner et al., 2005; Xie et al., 2007). Also, Low Density Lipid (LDL) receptor‐knockout (KO) mice, db/db mice, and TallyHo mice have been developed to study the effects of type II diabetes and obesity (Didion et al., 2007; Leong et al., 2015; Luttrell et al., 2008; Staprans et al., 2000), which are associated with erectile dysfunction (Burnett, 2006). We found that TMEM16A was expressed in murine CCSMC and that two different TMEM16A inhibitors (CACC(inh)‐A01 and Ani 9) blocked spontaneous Ca^2+^‐activated Cl^−^ currents in these cells. In addition, contractions of CCSM, evoked using phenylephrine, were inhibited by Ani9, suggesting that these channels play an important role in the regulation of CCSM contraction and therefore in the maintenance of penile detumescence.

## 2 MATERIALS AND METHODS

### 2.1 Tissue dissection

All procedures were carried out in accordance with the current European Union legislation and with the approval of Dundalk Institute of Technology Animal Use and Care Committee.

Mouse corpus cavernosum was chosen for the present study, as it has been widely used as a model for normal corpus cavernosum function and erectile dysfunction in normal and genetically manipulated animals (Luttrell et al., 2008; Werner et al., 2005). C57BL/6 mice were bred in‐house and housed in conventional cages, maximum 6 per cage with wood shavings for bedding. Male mice aged between 10–18 weeks old, weighing 25–30 g, were euthanized via intraperitoneal injection of sodium pentobarbital (100 mg.kg^−1^) and the penis was carefully removed from the pelvic bone before being placed in oxygenated Krebs solution. Surrounding tissue, including skeletal muscle, connective tissues, the penile bulb and corpus spongiosum, were trimmed by sharp dissection to expose the proximal corpus cavernosum. The crura were then excised and opened at the proximal end to expose the inner muscle of the corpus cavernosum. For experiments using urinary bladder smooth muscle, the bladder was removed from the animal and placed in Krebs solution. The bladder was opened along from the ureter end to the dome and pinned flat into a sheet. The urothelium was then carefully peeled away using fine forceps. To isolate segments of jejunum, the entire small intestine was removed and placed in Krebs solution. A small section of jejunum (2 cm) was cut away and pins were inserted into the mesentery along the axis of the tissues. The tissue was then opened in a longitudinal direction and the mucosal layer was removed.

### 2.2 Isometric tension recording

Crural sections of the corpus cavernosum ∼3 mm in length and 1.5 mm in diameter were removed. The sections were hooked transversely through the tunica albuginea, and one side of the tissue was cut open, exposing the tunica albuginea that was connected by the inner smooth muscle. The tissue sections were then mounted in organ baths containing Krebs (solution 1) at 37°C constantly bubbled with 95% O_2_ and 5% CO_2_ throughout the experiments. Tissue sections were adjusted to a tension of 4–6 mN and left to equilibrate for 45 min before experimentation. Tension was measured via transducers powered by a SI‐BAM21‐LCB Force Transducer Amplifier (World Precision Instruments, Europe), and recorded via Data‐Trax 2 software (World Precision Instruments, Europe) on a PC.

### 2.3 Single‐cell isolation

Isolated corpus cavernosum crura and detrusor muscle were cut into small pieces and placed in Hanks Ca^2+^‐free solution. Single CCSM cells and single detrusor SM cells were isolated using a collagenase‐proteinase mixture consisting of (per 5 ml of Hanks Ca^2+^‐free solution) 15 mg of collagenase (type IA, Sigma), 1 mg of proteinase (type XXIV, Sigma), 10 mg of BSA (Sigma), and 10 mg of trypsin inhibitor (Sigma). Tissue pieces were placed in the collagenase‐proteinase mixture for 5 min at 37°C and then transferred to Hanks Ca^2+^‐free solution and stirred for 5 min to release single smooth muscle cells. Cells were plated in Petri dishes containing Hanks Ca^2+^‐free solution (with added Ca^2+^ to a concentration of 100 μM) and stored at 4°C for use within 6 h.

### 2.4 Patch‐clamp recording

Recordings were made using the amphotericin B perforated patch method (Rae et al., 1991), where electrical access between the pipette and cell interior is achieved by inclusion of the pore forming compound amphotericin B (600 μg/mL) in the pipette solution (Hannigan et al., 2017).

Pipettes were pulled from borosilicate glass capillary tubing (1.5 mm OD, 1.17 mm ID; Harvard Apparatus, UK) to a tip of diameter of 1–1.5 μm and resistance of 2–3 MΩ. Voltage‐clamp commands were delivered via a Axopatch 1D (Molecular Devices) amplifier interfaced to a computer running pCLAMP software (Molecular Devices).

### 2.5 Immunocytochemistry

After dispersal, cells were left at room temperature for 90 min to adhere to the bottom of the dish. They were then incubated in 2% paraformaldehyde for 45 min at 4°C, before being washed three times with single strength Dulbecco's Phosphate Buffered Saline (1 × DPBS). Cells were then permeabilized with 0.3% Triton X‐100, and blocked with donkey serum for 10 min, before being washed three times with 1 × DPBS. Cells were incubated in anti‐TMEM16A antibody (rabbit polyclonal anti‐human, 1:200 dilution, Alomone Labs Cat# ab72984), or anti‐smooth muscle myosin heavy chain 11 antibody (rabbit polyclonal anti‐mouse, 1:200 dilution, Abcam Cat# ab53219, RRID:AB_2147146) for 24 h at 4°C and then washed three times with 1 × DPBS. This was followed by incubation of cells in secondary antibody conjugated to Alexa Fluor™ 488 (donkey polyclonal anti‐rabbit antibody, 1:1000 dilution, Molecular Probes Cat# A‐21206, RRID:AB_2535792) for 1 h at 4°C, and then washed with 1 × DPBS for 30 min with a solution change every 5 min. Controls were prepared simultaneously. Secondary antibody‐only controls, which omitted the primary antibody incubation, were processed and imaged with the same parameters as each experimental image.

### 2.6 Immunohistochemistry

The protocol was similar to immunocytochemistry protocol, except that the tissue was incubated in paraformaldehyde for 40 min at room temperature. The only primary antibody used was the anti‐TMEM16A antibody (rabbit polyclonal anti‐human, 1:200 dilution, Alomone Labs Cat# ab72984).

### 2.7 Imaging

Immunofluorescence of CCSM cells, bladder SM cells, and jejunum tissue was imaged using an Axioskop 2 LSM 510 Meta confocal microscope (Zeiss, Germany). A water‐dipping ×20 objective lens was lowered into the dish and cells were focused in transmitted light. Laser excitation (488 nm) was used to visualize immunoreactivity in the cells and the emission filter used was 505–530 nm. Confocal micrographs were generated from Z‐series scans taken at 0.3 μm depth intervals. Final images were constructed using the Z‐stack function within Image J software (Rasband, 2011).

### 2.8 Materials

Collagenase (Sigma), bovine serum albumin (Sigma), trypsin inhibitor (Sigma), 2‐aminoethoxydiphenyl borate (2APB, Sigma), amphotericin B (Sigma), tetracaine (Sigma), (R)‐(−)‐phenylephrine hydrochloride (Sigma), nifedipine (Tocris), (4‐Chloro‐2‐methylphenoxy)‐acetic acid [(2‐methoxyphenyl)methylene]hydrazide,2‐(4‐Chloro‐2‐methylphenoxy)‐N‐[(2 methoxyphenyl)methylideneamino]‐acetamide [Ani9] (Tocris), 6‐(1,1‐Dimethylethyl)‐2‐[(2‐furanylcarbonyl)amino]‐4,5,6,7‐tetrahydro‐benzo[b]thiophene‐3‐carboxylic acid [CaCCinh‐A01] (Tocris), cyclopiazonic acid (CPA, Abcam), GSK‐7975A (Merck).

The following solutions were used:

1. Krebs (in mM: 120 NaCl, 5.9 KCl, 25 NaHCO_3_, 5.5 Glucose, 1.2 NaH_2_PO_4_, 1.2 MgCl_2_, 2.5 CaCl_2_.2H_2_O, pH adjusted to 7.4 by bubbling with 95% O_2_ and 5% CO_2_).
2. Ca^2+^‐free Hanks' for cell isolation (in mM: 125 NaCl, 5.36 KCl, 10 Glucose, 2.9 Sucrose, 15.5 NaHCO_3_, 0.44 KH_2_PO_4_, 0.33 Na_2_HPO_4_, 10 HEPES, pH adjusted to 7.4 with NaOH).
3. Hanks (in mM: 125 NaCl, 5.36 KCl, 10 Glucose, 2.9 Sucrose, 4.17 NaHCO_3_, 0.44 KH_2_PO_4_, 0.33 Na_2_HPO_4_, 0.5 MgCl_2_.6H_2_O, 1.8 CaCl_2_.2H_2_O, 0.4 MgSO_4_.7H_2_O, 10 HEPES, pH adjusted to 7.4 with NaOH).
4. Low Cl^−^ Hanks (in mM: 86.0 Na glutamate, 39 NaCl, 5.36 KCl, 10 Glucose, 2.9 Sucrose, 4.17 NaHCO_3_, 0.44 KH_2_PO_4_, 0.33 Na_2_HPO_4_, 0.5 MgCl_2_.6H_2_O, 1.8 CaCl_2_.2H_2_O, 0.4 MgSO_4_.7H_2_O, 10 HEPES, pH adjusted to 7.4 with NaOH).
5. Cs^+^ whole cell perforated patch solution (E_Cl_ = 0, when bath solution was Hanks) (in mM: 133 CsCl, 1 MgCl_2_.6H_2_O, 0.5 EGTA, 10 HEPES, pH adjusted to 7.2 with CsOH).
6. 1× Dulbecco's Phosphate Buffered Saline (DPBS) purchased from Thermofisher Scientific Cat# 14190094.
7. 2% Paraformaldehyde (PFA): 2 g of PFA (Sigma), 35 ml 5 M NaOH, 65 ml 1 × DPBS (heated to 60°C).

### 2.9 Statistics and data analysis

All statistical analyses and curve fitting were performed using GraphPadPrism version 7.0 e (GraphPad Software, Inc.) Values are means ± SE; *n* is the number of animals which equalled the number of cells or tissue strips. Hence, all ‘*n*’ values quoted refer to individual cells (electrophysiology) or tissue strips (tension) taken from different animals. For data requiring statistical comparisons (electrophysiology and tension) a sample size of *n* = 6 was chosen, based on previous power calculations and extensive experience. Immunocytochemistry data were not subjected to statistical analysis and were replicated in 3 separate animals. Statistical comparisons were made using Student's paired *t*‐test on raw (non‐normalized) data. Multiple comparisons were performed using one‐way ANOVA for repeated measures with the Dunnett or Tukey post hoc test. *p* < 0.05 was regarded as statistically significant. Electrophysiological data were analyzed by the operator using pCLAMP software (Molecular Devices) to electronically measure either maximal responses or at predetermined time points, as specified in the results.

## 3 RESULTS

### 3.1 Expression of TMEM16A in CCSM cells

**FIGURE 1:**
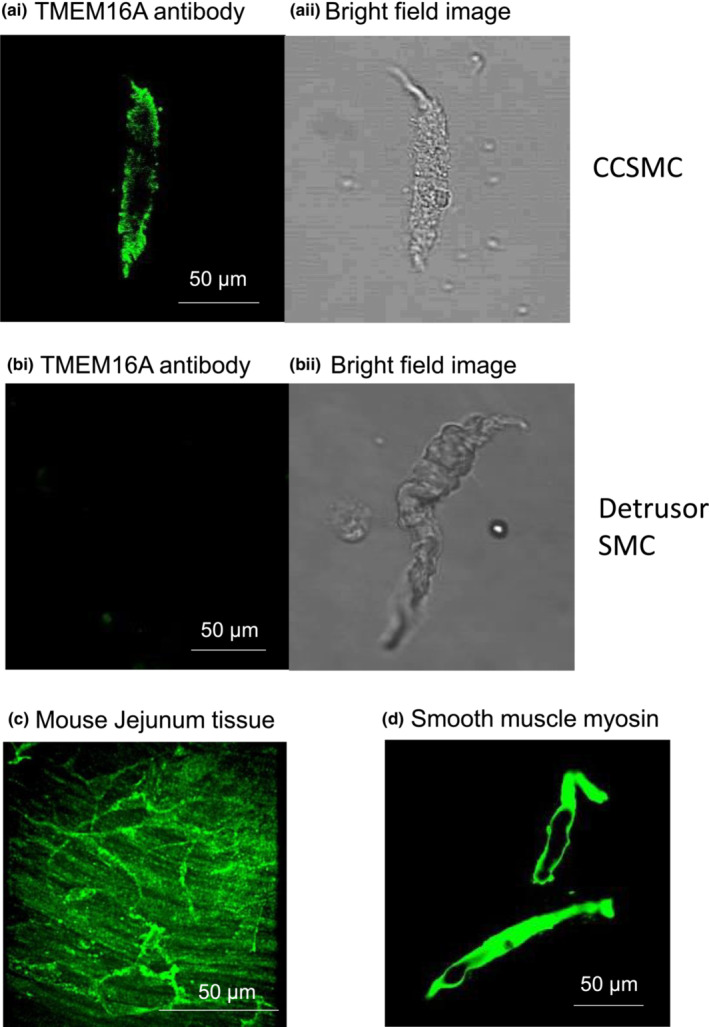
Immunofluorescence detection of TMEM16A in freshly isolated CCSM cells. (ai) representative confocal photomicrograph showing that freshly isolated mouse CCSM cells were immunoreactive to a selective TMEM16A antibody, demonstrated by the strong immunofluorescence signal detected [green] (*n* = 3 from 3 animals). (aii) bright field image of the same cell as in ai. (bi) negative control, representative confocal photomicrograph showing no immunofluorescence detected in detrusor SM cell exposed to the TMEM16A antibody (*n* = 3 from 3 animals). (bii) bright field image of the same cell as in bi. (c) Positive control, representative confocal photomicrographs showing robust TMEM16A staining in mouse jejunum tissue strip (*n* = 3 from 3 animals). (d) Smooth muscle cells were identified by smooth muscle myosin positive immunoreactivity [green] (*n* = 3 from  3 animals)

The representative confocal photomicrographs in Figure 1a confirm that freshly isolated CCSM cells were immunoreactive for TMEM16A, as demonstrated by the strong immunofluorescence in cells treated with a TMEM16A antibody (Figure 1ai). Staining shown in Figure 1ai was similar in three procedures carried out on cells dispersed from three animals. Figure 1aii shows a bright field image of the same cell as in Figure 1ai. The TMEM16A antibody was validated with negative and positive controls. Figure 1bi shows a negative control, demonstrating that immunofluorescence was not observed in the vast majority of murine detrusor SM cells, while weak immunofluorescence was observed in <2% of the detrusor cells. Figure 1bii shows a bright field image of the same cell as in Figure 1bi. Since interstitial cells of Cajal (ICC) in murine jejunum express TMEM16A proteins (Gomez‐Pinilla et al., 2009), we used the same antibody on jejunum smooth muscle tissue strips as a positive control. Figure 1c shows immunofluorescence on myenteric ICC located between the longitudinal and circular muscle layers of the jejunum. We also carried out staining for smooth muscle myosin (SMM) heavy chain 11 in CCSM cells. In this case, dense staining was observed throughout the cells (Figure 1d). Therefore, spindle shaped cells dispersed from mouse CCSM were immunoreactive to SMM heavy chain and TMEM16A antibodies, confirming the expression of TMEM16A in CCSM cells.

### 3.2 Two different TMEM16A‐specific blockers inhibited chloride tail currents

**FIGURE 2:**
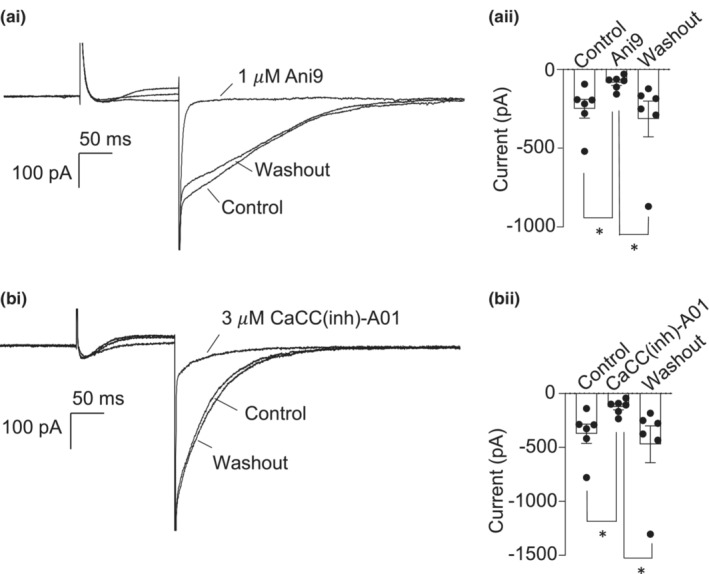
Two different TMEM16A‐specific blockers inhibited chloride tail currents. (ai) the cell was held at −60 mV and stepped to 0 mV. It was then stepped down to −80 mV to evoke a tail current. Representative traces showing the reversible effect of 1 μM Ani9 on tail current. (aii) summary data showing the effect of 1 μM Ani9 on tail current (*n* = 6 cells from 6 animals, *p < 0.05; one way ANOVA). (bi) representative traces showing the reversible effect of 3 μM CaCC(inh)‐A01 on tail current. (bii) summary data showing the effect of 3 μM CaCC(inh)‐A01 on tail current (*n* = 6 cells from 6 animals, *p < 0.05; one way ANOVA)

Having confirmed the expression of TMEM16A in mouse CCSM, we next evaluated the effects of specific TMEM16A antagonists on CCSM electrical activity. First, we examined the effects of the TMEM16A antagonists, Ani9 and CaCC(inh)‐A01, on evoked tail current in isolated CCSM cells using patch clamp techniques. Cells were held at −60 mV and currents were evoked by a step from −60 mV to 0 mV. Tail currents were then evoked by a step down to −80 mV for 600 ms. Figure 2ai,bi are representative traces which show that 1 μM Ani9 and 3 μM CaCC(inh)‐A01 inhibited tail current amplitude, suggesting that the tail current was conducted through TMEM16A channels. Summary data for the effects of the two TMEM16A blockers on peak tail current amplitude are shown in Figure 2aii,bii.

### 3.3 Effect of low Cl^−^ hanks on tail currents

**FIGURE 3:**
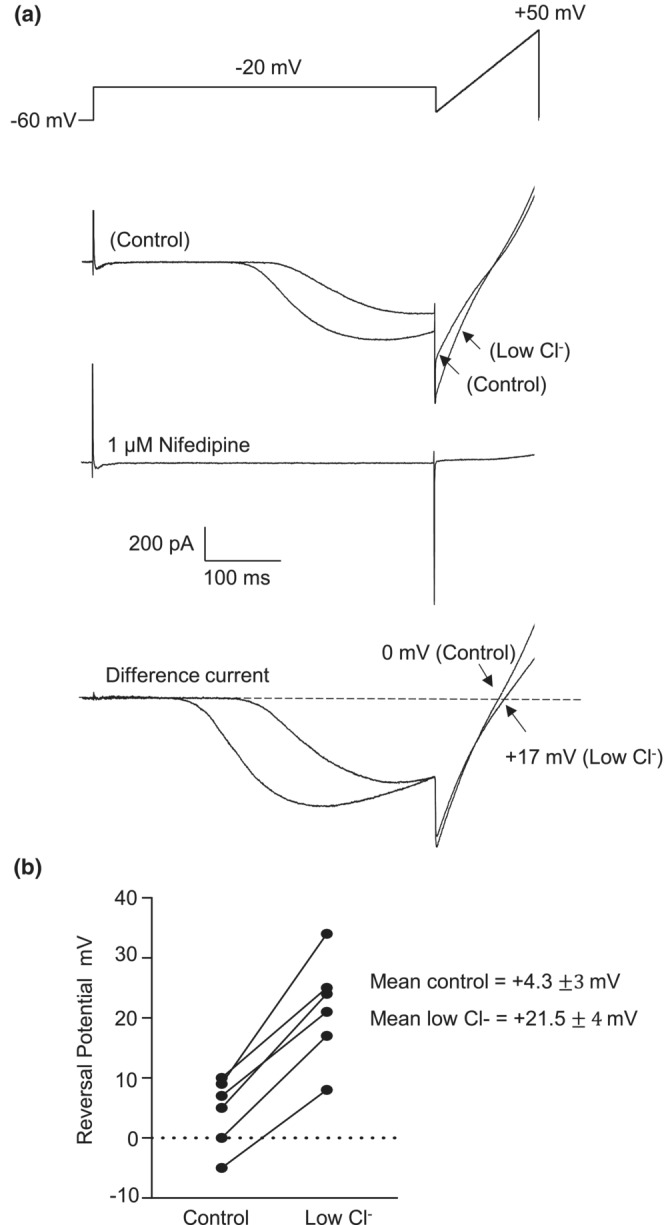
Effect of low Cl^−^ hanks buffer on tail currents. (a) an isolated CCSMC was held at −60 mV and stepped to 0 mV followed by a voltage ramp from −50 to +50 mV. Recordings were made in 135 mM external Cl^−^ (control) and 49 mM external Cl^−^ (low Cl^−^) solutions. Capacitive current in all data presented was eliminated by subtracting from traces evoked in the presence of nifedipine, revealing the difference current. (b) the shift in the reversal potential of the tail current is consistent with the current being carried through Ca^2+^‐activated Cl^−^ channels (*n* = 6 cells from 6 animals; *p < 0.05; paired *t*‐test)

Next, to confirm that the tail current was carried by chloride ions, we examined the effect of reducing the external Cl^−^ concentration on tail current reversal potential. Cells were initially stepped to a test potential of −20 mV for 500 ms followed by a 150 ms voltage ramp from −50 to +50 (Figure 3a). The step duration was sometimes varied depending on the rate of current activation. The procedure was then repeated a few seconds after reducing [Cl]_o_ to 49 mM. This had the effect of shifting the reversal potential of the tail current in the positive direction. Both sweeps were then corrected for capacitance and leakage currents by subtracting a ‘null’ trace evoked in the presence of nifedipine. Nifedipine was previously shown to abolish Ca^2+^‐activated Cl^−^ currents evoked by voltage steps by blocking Ca^2+^ influx via L‐type Ca^2+^ channels (Craven et al., 2004). In control (135 mM external Cl^−^, calculated E_Cl_ = 0 mV) the difference current reversed at 0 mV (+3 mV when corrected for a junction potential of +3 mV), while in low external Cl^−^ solution (49 mM external Cl^−^, calculated E_Cl_ = +27 mV) it shifted to +17 mV (+19 mV when corrected for +2 mv junction potential). In the example shown in Figure 3a, the amplitude of the inward current during the step to −20 mV also increased in the presence of low external Cl^−^. In six experiments, the mean reversal potential was +1 mV for 135 mM external Cl^−^ and + 24 mV for 49 mM external Cl^−^ (corrected for junction potentials, *p* < 0.05, Figure 3b). Based on the reversal of tail current at the Cl^−^ equilibrium potential, and its susceptibility to external Cl^−^ concentration, it is very likely that the tail currents were mainly carried by chloride ions.

### 3.4 Two different TMEM16A‐specific blockers abolished STICs and STDs

**FIGURE 4:**
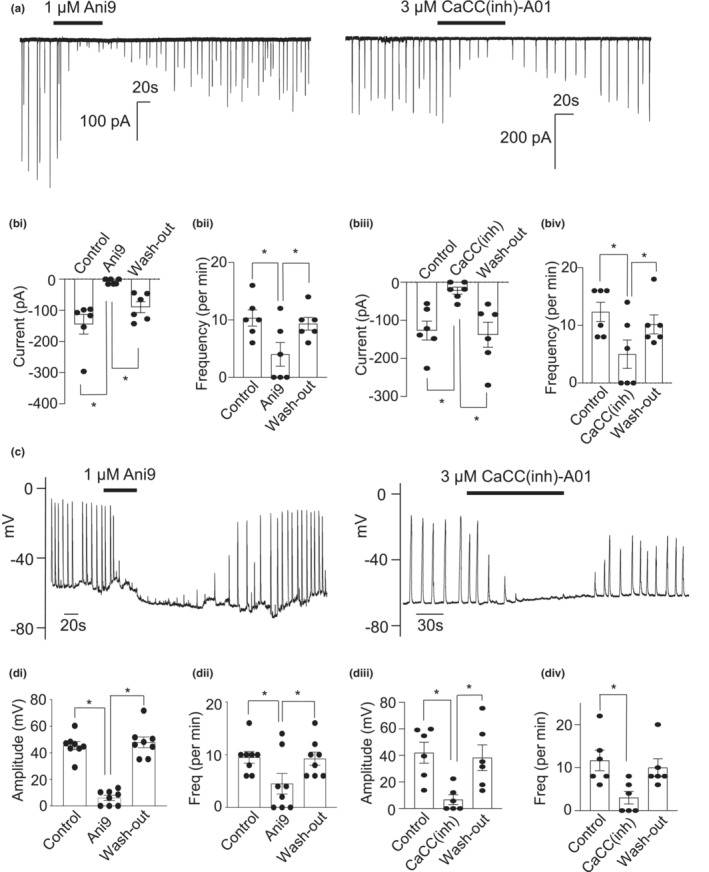
Two different TMEM16A‐specific blockers abolished STICs and STDs. (a) an example showing the effect of 1 μM Ani9 and 3 μM CaCC(inh)‐A01 on STICs held under voltage clamp at −60 mV. (bi) summary of the effect of 1 μM Ani9 on STIC amplitude (*n* = 6 cells from 6 animals; *p < 0.05; one way ANOVA). (bii) summary of the effect of 1 μM Ani9 on STIC frequency (*n* = 6 cells from 6 animals; *p < 0.05; one way ANOVA). (biii) summary of the effect of 3 μM CaCC(inh)‐A01 on STIC amplitude (*n* = 6 cells from 6 animals; *p < 0.05; one way ANOVA). (biv) summary of the effect of 3 μM CaCC(inh)‐A01 on STIC frequency (*n* = 6 cells from 6 animals; *p < 0.05; one way ANOVA). (c) an example showing the effect of 1 μM Ani9 and 3 μM CaCC(inh)‐A01 on STDs in current clamp mode. (di) summary of the effect of 1 μM Ani9 on STD amplitude (*n* = 6 cells from 6 animals; *p < 0.05; one way ANOVA). (dii) summary of the effect of 1 μM Ani9 on STD frequency (*n* = 6 cells from 6 animals; *p < 0.05; one way ANOVA). (diii) summary of the effect of 3 μM CaCC(inh)‐A01 on STD amplitude (*n* = 6 cells from 6 animals; *p < 0.05; one way ANOVA). (div) summary of the effect of 3 μM CaCC(inh)‐A01 on STD frequency (*n* = 6 cells from 6 animals; *p < 0.05; one way ANOVA)

**FIGURE 5:**
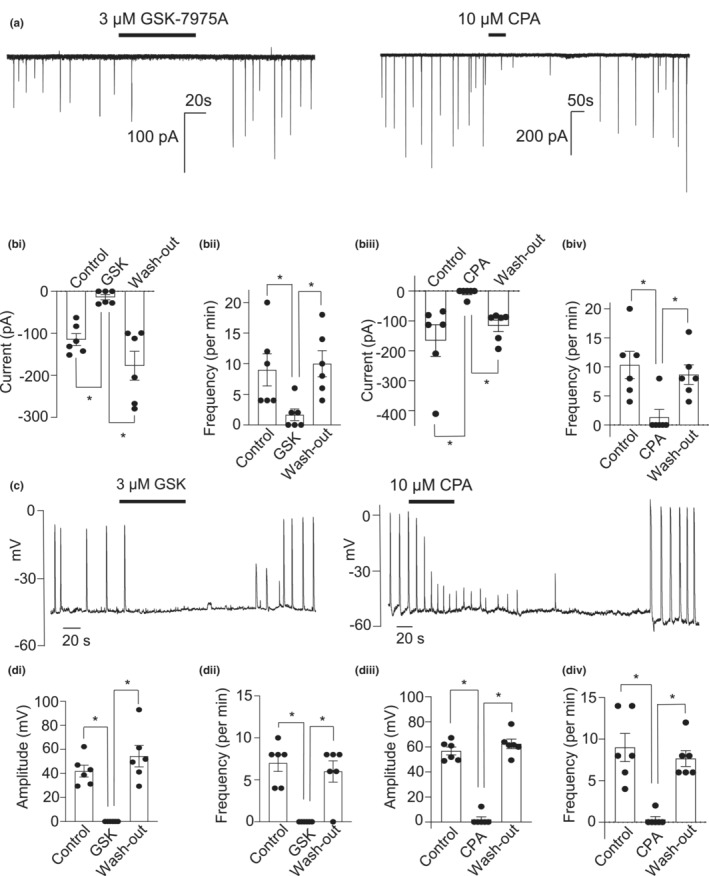
Involvement of CRAC channels and Ca^2+^ stores in activation of STICs and STDs. (a) an example showing the effect of 3 μM GSK7975A and 10 μM CPA on STICs held under voltage clamp at −60 mV. (bi) summary of the effect of 3 μM GSK7975A on STICs amplitude (*n* = 6 cells from 6 animals; *p < 0.05; one way ANOVA). (bii) summary of the effect of 3 μM GSK7975A on STIC frequency (*n* = 6 cells from 6 animals; *p < 0.05; one way ANOVA). (biii) summary of the effect of 10 μM CPA on STICs amplitude (*n* = 6 cells from 6 animals; *p < 0.05; one way ANOVA). (Biv) summary of the effect of 10 μM CPA on STIC frequency (*n* = 6 cells from 6 animals; *p < 0.05; one way ANOVA). (c) an example showing the effect of 3 μM GSK7975A and 10 μM CPA on STDs in current clamp mode. (di) summary of the effect of 3 μM GSK7975A on STD amplitude (*n* = 6 cells from 6 animals; *p < 0.05; one way ANOVA). (dii) summary of the effect of 3 μM GSK7975A on STD frequency (*n* = 6 cells from 6 animals; *p < 0.05; one way ANOVA). (diii) summary of the effect of 10 μM CPA on STD amplitude (*n* = 6 cells from 6 animals; *p < 0.05; one way ANOVA). (div) summary of the effect of 10 μM CPA on STD frequency (*n* = 6 cells from 6 animals; *p < 0.05; one way ANOVA)

**FIGURE 6:**
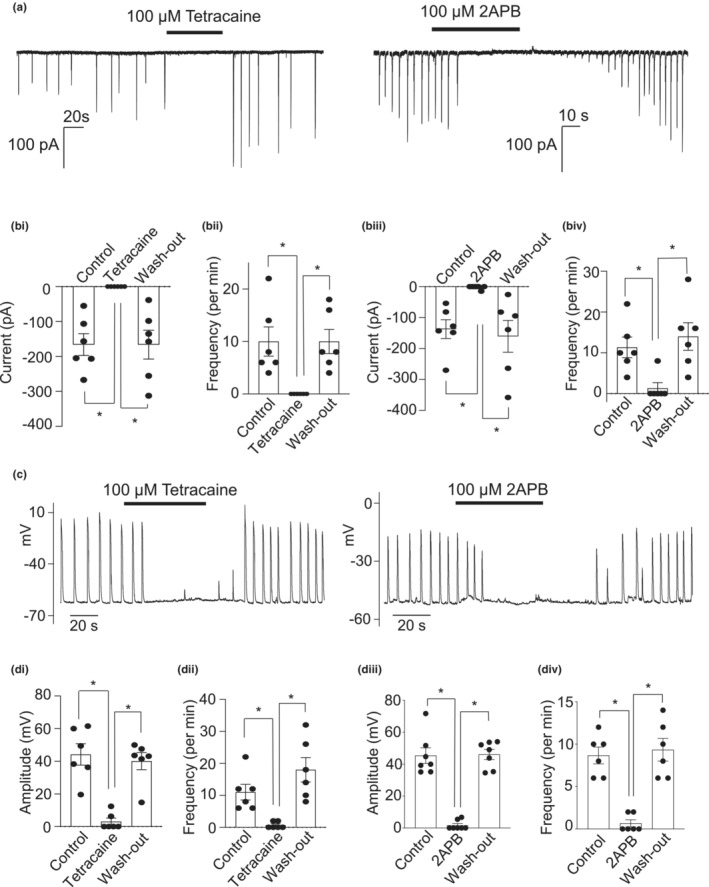
Involvement of both RyR and IP_3_R in activation of STICs and STDs. (a) an example showing the effect of 100 μM tetracaine and 100 μM 2APB on STICs held under voltage clamp at −60 mV. (bi) summary of the effect of 100 μM tetracaine on STIC amplitude (*n* = 6 cells from 6 animals; *p < 0.05; one way ANOVA). (bii) summary of the effect of 100 μM tetracaine on STIC amplitude (*n* = 6 cells from 6 animals; *p < 0.05; one way ANOVA). (biii) summary of the effect of 100 μM 2APB on STIC frequency (*n* = 6 cells from 6 animals; *p < 0.05; one way ANOVA). (biv) summary of the effect of 100 μM 2APB on STIC amplitude (*n* = 6 cells from 6 animals; *p < 0.05; one way ANOVA). (c) an example showing the effect of 100 μM tetracaine and 100 μM 2APB on STDs in current clamp mode. (di) summary of the effect of 100 μM tetracaine on STD amplitude (*n* = 6 cells from 6 animals; *p < 0.05; one way ANOVA). (dii) summary of the effect of 100 μM tetracaine on STD frequency (*n* = 6 cells from 6 animals; *p < 0.05; one way ANOVA). (diii) summary of the effect of 100 μM 2APB on STD amplitude (*n* = 6 cells from 6 animals; *p < 0.05; one way ANOVA). (div) summary of the effect of 100 μM 2APB on STD frequency (*n* = 6 cells from 6 animals; *p < 0.05; one way ANOVA)

**FIGURE 7:**
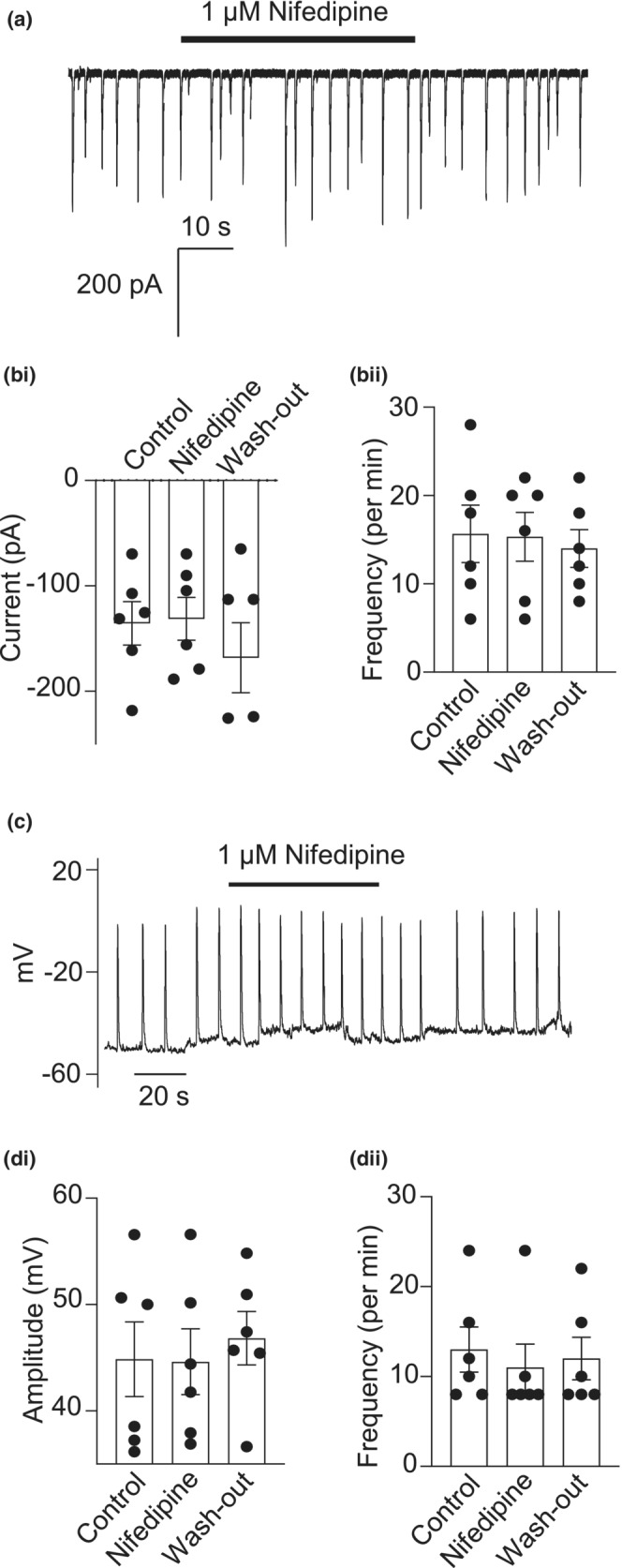
Involvement of L‐type Ca^2+^ channel in activation of STICs and STDs. (a) an example showing the effect of 1 μM nifedipine on STICs held under voltage clamp at −60 mV. (bi) summary of the effect of 1 μM nifedipine on STIC amplitude (*n* = 6 cells from 6 animals; *p > 0.05; one way ANOVA). (bii) summary of the effect of 1 μM nifedipine on STIC frequency (*n* = 6 cells from 6 animals; *p > 0.05; one way ANOVA). (c) an example showing the effect of 1 μM nifedipine on STDs in current clamp mode. (di) summary of the effect of 1 μM nifedipine on STD amplitude (*n* = 6 cells from 6 animals; *p > 0.05; one way ANOVA). (dii) summary of the effect of 1 μM nifedipine on STD frequency (*n* = 6 cells from 6 animals; *p > 0.05; one way ANOVA)

In voltage‐clamp mode, 141 out of 298 (47%) mouse CCSM cells studied exhibited spontaneous transient inward currents (STICs) when held at −60 mV. To determine if STICs were carried by Ca^2+^‐activated Cl^−^ currents, the effects of two different TMEM16A‐specific blockers, Ani9 and CaCC(inh)‐A01, were tested on these spontaneous currents (Figure 4a). For the purposes of summarizing these data, average STIC amplitude in each cell was calculated for the 30 s period immediately before application of each drug, and again for 30 s after the drug had exerted its maximal effect. The average values were then used to calculate the means represented in Figures 4, 5, 6, 7, and 9.

Ani9 and CaCC(inh)‐A01 both greatly reduced STIC amplitude, but had less effect on frequency (Figure 4a). Overall, Ani9 (1 μM) and CaCC(inh)‐A01 (3 μM) reduced mean STIC amplitude by 95% and 83%, respectively (Figure 4bi,biii) and frequency by 61% and 60%, respectively (Figure 4bii,biv).

Cells were also studied in current clamp mode to observe changes in membrane potential. In these experiments, the resting membrane potential was adjusted to between −50 mV and − 60 mV by injecting small hyperpolarizing current. The majority of cells (72 of 111) displayed regular depolarizations of membrane potential, referred to as spontaneous transient depolarizations (STDs). These STDs were sensitive to Ani9 and CaCC(inh)‐A01 (Figure 4C). 1 μM Ani9 reduced STD amplitude by 87% and frequency by 53% (Figure 4di,dii), while 3 μM CaCC(inh)‐A01 reduced STD amplitude by 84% and frequency by 74% (Figure 4diii,div).

### 3.5 Contribution of Ca^2+^ stores to STICs and STDs in CCSMC

Ca^2+^‐release‐activated Ca^2+^ (CRAC) channels are known to be involved in SR refilling (Putney et al., 2017). We examined if CRAC channels were involved in the generation of STICs and STDs in CCSM cells by examining the effects of GSK7975A (3 μM), an ORAI channel blocker. GSK7975A had a marked inhibitory effect on STICs (Figure 5a) and STDs (Figure 5c) in CCSM cells. Summary data showing the effect of GSK7975A on STICs and STDs (*n* = 6) are presented in Figure 5bi,bii,di,dii, respectively. The effect of cyclopiazonic acid (CPA, 10 μM), a blocker of the sarcoplasmic Ca^2+^‐ATPase, was also studied on STICs and STDs. CPA abolished these events (Figure 5a,c), while in 1 cell out of 6 it also caused a depolarization. Summary data for the effect of CPA on STICs and STDs are summarized in Figure 5biii,biv,diii,div, respectively.

### 3.6 Involvement of both RyR and IP_3_R in activation of STICs and STDs

As the previous experiments suggested that Ca^2+^ stores are essential for the generation of STICs and STDs, we tested the relative contributions of RyRs and IP_3_Rs to mouse CCSM electrical activity using tetracaine, an inhibitor of RyRs (Laver & van Helden, 2011) and 2APB, a blocker of IP_3_Rs(Maruyama et al., 1997). Figure 6a shows an example where tetracaine (100 μM) and 2APB (100 μM) reversibly inhibited STICs. Summary data for these effects are shown in Figure 6b. Tetracaine abolished STICs, while 2APB reduced STIC amplitude by 98% amplitude and frequency by 88%. Tetracaine and 2APB also reversibly blocked STDs (Figure 6c,d), as evidenced by the 93% reduction in amplitude and 94% decrease in frequency with tetracaine, 96% block in amplitude and 92% reduction in frequency with 2APB. Taken together, these results suggest that Ca^2+^ release from both RyRs and IP_3_Rs are required for STICs and STDs in CCSM cells.

### 3.7 Involvement of L‐type Ca^2+^ channel in activation of STICs and STDs

**FIGURE 8:**
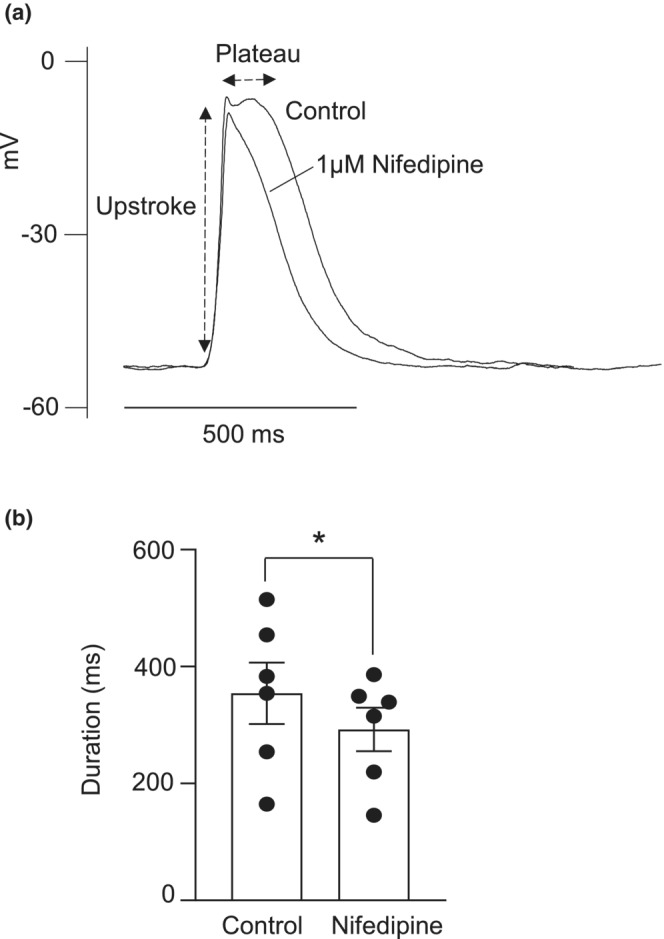
Involvement of L‐type Ca^2+^ channel in the plateau phase of STD. (a) 1 μM nifedipine reduced the duration of STDs in CCSM. (b) Summary data showing the effect of 1 μM nifedipine on the mean duration of STDs (*n* = 6 cells from 6 animals; *p < 0.05; paired *t*‐test)

To find out if Ca^2+^ entry through L‐type Ca^2+^ channels was involved in the activation of STICs or STDs, the effect of nifedipine was tested on these events. Nifedipine (1 μM) failed to diminish the amplitude or frequency of STICs (Figure 7a,b) or STDs (Figure 7c,d). However, it was apparent that the mean duration of the STDs was reduced (Figure 8a). Summary data in Figure 8b shows that the mean duration of depolarizations (time from half maximum voltage to half minimum voltage) was reduced in the presence of 1 μM nifedipine.

### 3.8 Ani9 inhibited phenylephrine‐induced STICs and phenylephrine‐induced STDs

**FIGURE 9:**
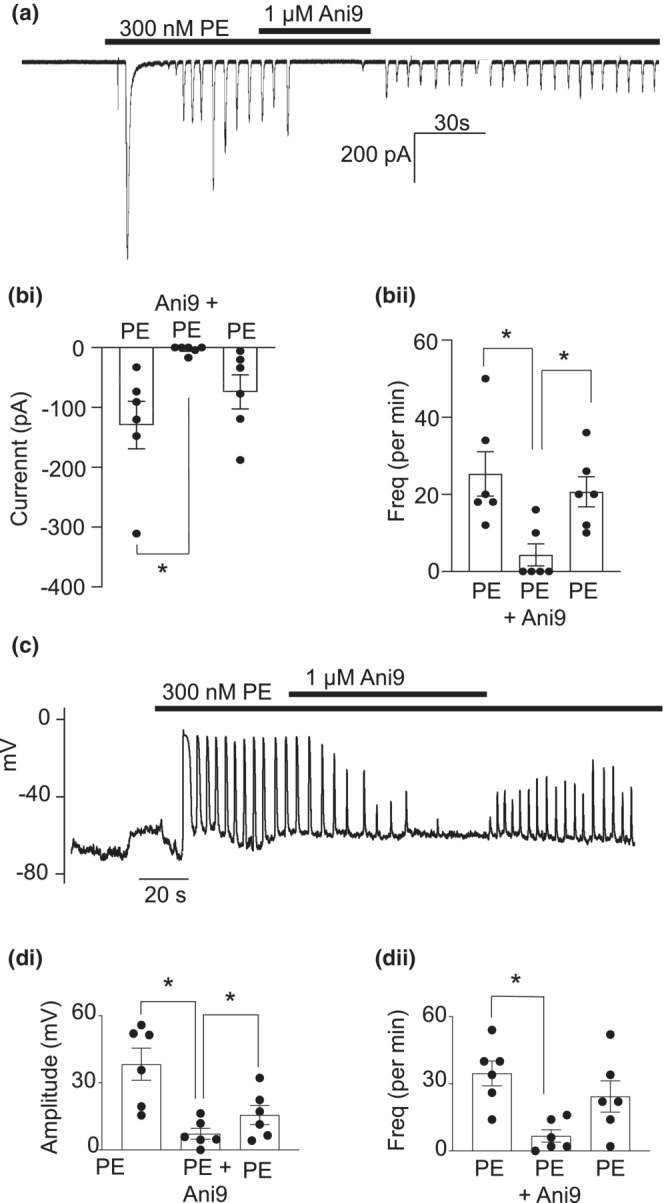
Ani9 inhibited phenylephrine‐induced transient inward currents and transient depolarizations. (a) 300 nM phenylephrine evoked a large, transient inward current, followed by STICs which were susceptible to blockade by 1 μM Ani9. There was only partial return of phenylephrine response after washout from Ani9 (bi) summary data showing the effect of Ani9 on the amplitude (*n* = 6 cells from 6 animals; *p < 0.05; one way ANOVA) and (bii) frequency of phenylephrine induced depolarizations (*n* = 6 cells from 6 animals; *p < 0.05; one way ANOVA). (c) 300 nM phenylephrine evoked regular, oscillating depolarizations, which were susceptible to block by 1 μM Ani9. (di) summary data showing the effect of Ani9 on the amplitude (*n* = 6 cells from 6 animals; *p < 0.05; one way ANOVA) and (dii) frequency of phenylephrine induced depolarizations (*n* = 6 cells from 6 animals; *p < 0.05; one way ANOVA)

Since the main excitatory innervation in CC is adrenergic, it was of interest to examine if TMEM16A contributed to adrenergic responses in CCSM cells. In voltage‐clamped CCSM cells, addition of the α_1_‐adrenoceptor agonist, phenylephrine (300 nM), caused a large transient inward current, followed by smaller, oscillating transient inward currents (Figure 9a). Addition of Ani9 (1 μM) significantly reduced both the amplitude (Figure 9a,bi) and frequency (Figure 9a,bii) of the oscillating currents. The effect of phenylephrine was partially restored upon wash‐out (Figure 9a,b). In current clamp mode, 300 nM phenylephrine evoked a series of oscillating depolarizations (Figure 9c) that were susceptible to blockade by 1 μM Ani9 (Figure 9c,di,dii). These data suggest that TMEM16A may be involved in excitatory adrenergic responses in CCSM cells.

### 3.9 Ani9 reduced the frequency of phenylephrine‐induced contractions in CCSM tissue strips

**FIGURE 10:**
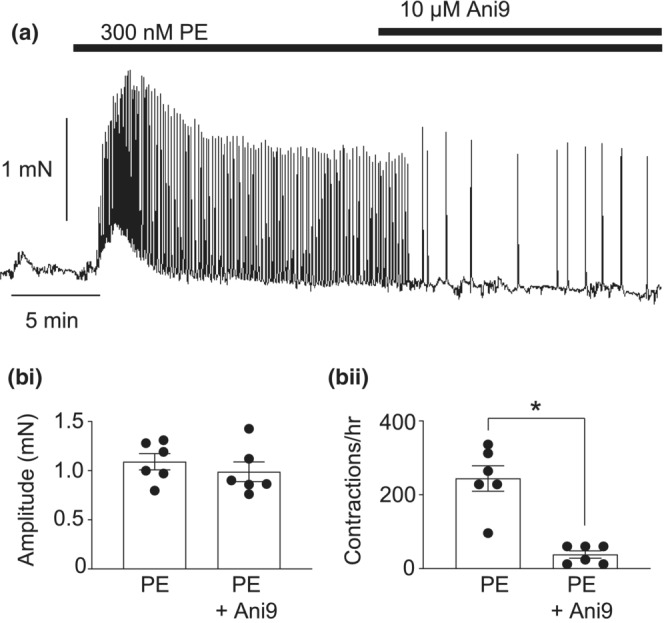
Ani9 reduced the frequency of phenylephrine‐induced contractions in CCSM tissue strips. (a) Representative trace showing phasic, oscillatory contractions evoked by 300 nM phenylephrine that are susceptible to blockade by 10 μM Ani9. (bi) summary data showing the effect of 10 μM Ani9 on mean evoked contraction amplitude (*n* = 6 strips from 6 animals; *p > 0.05; paired *t*‐test) and (Bii) evoked contraction events per hour (*n* = 6 strips from 6 animals; *p < 0.05; paired *t*‐test)

To examine the functional role of TMEM16A channels in CCSM contractility we examined if phenylephrine‐induced contractions of CCSM strips were affected by Ani9. The representative trace in Figure 10a shows that phenylephrine induced a series of phasic, oscillatory contractions. Ani9 (10 μM) reduced the frequency, but not the amplitude, of these events (Figure 10a,bi,bii). Summary data in Figure 10bii shows that Ani9 significantly reduced the mean frequency of the phenylephrine‐induced contractions from 244 ± 35 to 38 ± 10 contractions per hour.

Since α‐adrenoceptor‐induced contractions in corpus cavernosum can be antagonized by L‐type Ca^2+^ channel blockers (McCloskey et al., 2009), it was important to ensure that Ani9 did not have a non‐specific blocking effect on these channels. Patch clamp experiments confirmed that Ani9 (10 μM) had very little effect on L‐type Ca^2+^ current (Figure S1). These experiments suggest that TMEM16A may have a functional role in adrenergic responses in corpus cavernosum tissue.

## 4 DISCUSSION

Ca^2+^‐activated Cl^−^ channels regulate many physiological functions, including cell excitability (Cotton et al., 1997), osmotic balance, and fluid secretion (Eggermont, 2004). In smooth muscle, activation of these channels is usually excitatory because the Cl^−^ equilibrium potential (E_Cl_) is more positive than the resting membrane potential. For example, in rabbit CCSM, the resting membrane potential is within the range − 50 mV to −41 mV (Hashtani et al., 2005) while in smooth muscles E_Cl_ is within the range − 38 to −19 mV (Aickin & Brading, 1982; Chipperfield & Harper, 2000). Consequently, opening of Cl^−^ channels in this tissue will induce depolarization and activate L‐type Ca^2+^ channels, resulting in Ca^2+^‐influx and contraction (Large & Wang, 1996). Intracellular recordings from corpus cavernosum show that the tissue develops spontaneous depolarizations that are blocked by niflumic acid, a Ca^2+^‐activated chloride blocker and dihydropyridines, blockers of L‐type Ca^2+^ channels (Hashitani et al., 2005).

TMEM16A was identified as a Ca^2+^‐activated chloride channel (CaCC) in 2008 (Caputo et al., 2008; Schroeder et al., 2008; Yang et al., 2008) and was proposed as the molecular identity of Ca^2+^‐activated Cl^−^ channels in rabbit CCSM (Hannigan et al., 2017). In the present paper, we present evidence based on immunocytochemistry, [Cl^−^]_o_‐dependence, and pharmacology, that TMEM16A is functionally expressed in mouse CCSM cells. Inhibition of chloride tail currents using two different Ca^2+^‐activated Cl^−^ channel blockers, combined with the shift in for the reversal potential of the tail current in ion substitution experiments, verified the presence of Ca^2+^‐activated Cl^−^ currents in isolated CC myocytes. Furthermore, the molecular identity of Ca^2+^‐activated Cl^−^ channels was confirmed to be TMEM16A via CCSM single‐cell immunofluorescence staining.

In the present study, both STICs and STDs in murine CCSMC were inhibited by CPA, consistent with a role for Ca^2+^ release from the SR in the generation of this activity (Craven et al., 2004). STICs and STDs were also abolished by tetracaine and 2APB, antagonists of ryanodine receptors (Rae et al., 1991) and IP_3_ receptors (Peppiatt et al., 2003), respectively. Therefore, it appears that the spontaneous activation of TMEM16A channels in murine CCSMC relies on a combination of Ca^2+^ release from both RyRs and IP_3_Rs. It should be noted however, that both tetracaine and 2‐APB may also have off‐target effects. For example, tetracaine can block Na_V_ channels (Li et al., 1999). Although Na_V_1.5 channels are present in mouse CCSM, most would be inactivated at the resting membrane potential (−50 mV to ‐60 V), providing little contribution to myogenic activity at these potentials, under normal circumstances (Eggermont, 2004). The IP_3_R inhibitor, 2APB has been shown to inhibit store operated Ca^2+^ (SOC) entry (Peinelt et al., 2006; Peppiatt et al., 2003). Unfortunately, almost every IP_3_R antagonist has limitations and their selectivity has been questioned. For example, heparin has low tissue penetration (Saleem et al., 2014) and Xestospongin C blocks L‐type Ca^2+^ channels and K^+^ channels (Ozaki et al., 2002), both of which contribute to CCSM phasic contractions (Hannigan et al., 2017; McCloskey et al., 2009). Although 2APB may have unwanted off‐target effects in some cell types, we previously found that it was able to inhibit IP_3_‐mediated Ca^2+^ signals (Drumm et al., 2015; Drumm et al., 2018; Griffin et al., 2016; Johnston et al., 2005; Morgan et al., 2022; Sergeant et al., 2001), without affecting caffeine‐induced Ca^2+^‐entry (Drumm et al., 2015), sarcoplasmic reticulum refilling (Sergeant et al., 2001), or capacitive Ca^2+^ entry (Bradley et al., 2005). Therefore, we chose 2APB as the antagonist of IP_3_Rs in our study.

Vascular diseases like diabetes and hypertension can impair neurogenic and endothelium‐mediated relaxation of penile smooth muscle (Ledda, 2000). A survey involving 7689 patients found that ED was prevalent (>50%) among those with diabetes and/or hypertension (Giuliano et al., 2004). Alterations in STIM and ORAI activity has been highlighted in injured or diseased blood vessels (Zhang et al., 2011), and it was shown that the transcriptional and protein expression, as well as the function of both STIM and ORAI were upregulated in the aorta of hypertensive rats (Giachini et al., 2009). Since vascular dysfunction can compromise cavernosal function (Burnett, 2006), we investigated the effect of GSK‐7975A, a selective store‐operated Ca^2+^ channel inhibitor, on STICs and STDs recorded from murine CCSM. A low concentration (3 μM) of GSK‐7975A inhibited these events, suggesting that they were reliant on store‐operated Ca^2+^ entry. In contrast, the L‐type Ca^2+^ channel blocker, nifedipine did not affect the amplitude and frequency of STICs or STDs, suggesting that Ca^2+^ entry via L‐type Ca^2+^ channels was not involved in the initiation of these events. However, nifedipine shortened the duration of the STDs, suggesting that influx via L‐type Ca^2+^ currents contributed to the STD plateau potential. This is similar to findings in sheep urethral SM cells, where nifedipine reduced STD plateau duration, but did not reduce STD frequency (Cotton et al., 1997). However, nifedipine also reduced STD amplitude, possibly as a result of larger L‐type Ca^2+^ currents expressed in these urethral cells (Cotton et al., 1997).

The functional role of TMEM16A in intact CCSM tissue strips was investigated using Ani9, a selective TMEM16A blocker (Seo et al., 2016). Phenylephrine (300 nM) induced phasic contractions, which were significantly disrupted by Ani9, suggesting a role for TMEM16A in the regulation of CCSM contractility by sympathetic nerves. Some TMEM16A blockers are known to also block L‐type Ca^2+^ current (Hannigan et al., 2017), hence it was important to check that the effects of Ani9 were not attributable to an off‐target effect on L‐type Ca^2+^ channels. We found that Ani9 (10 μM) had little effect on L‐type Ca^2+^ currents, therefore its inhibitory effect on phasic contractions of mouse CCSM tissue strips is not due to an effect on L‐type Ca^2+^ channels, as may be the case with other CACC blockers (Hannigan et al., 2017).

In conclusion, the present study provides direct evidence that the mouse CCSM exhibit spontaneous TMEM16A currents that are activated by Ca^2+^ released from the sarcoplasmic reticulum. Furthermore, these channels contribute to the contractile state of the CCSM and therefore are likely to help to maintain penile detumescence. We propose a basic scheme whereby spontaneous depolarizations are initiated by TMEM16A as a result of spontaneous Ca^2+^ release from stores that depends on both RyR and IP_3_R, while refilling of the stores depends on both the SERCA pump and external Ca^2+^ influx via STIM/Orai store operated Ca^2+^ entry. In turn, the depolarizations result in activation of L‐type Ca^2+^ currents, leading to voltage‐dependent Ca^2+^ influx and contraction. This underlying mechanism is upregulated upon α‐adrenoceptor stimulation, most likely via stimulation of phospholipase C (PLC) and IP_3_ generation, thus pushing the balance toward penile detumescence. TMEM16A channels in CCSMC could, therefore, be a potential new target to treat erectile dysfunction. Future studies should be directed toward examining the expression and effects of TMEM16A in mouse models of type II diabetes (e.g. db/db mice) that develop erectile dysfunction (Luttrell et al., 2008).

## AUTHOR CONTRIBUTIONS

Xin Rui Lim: conception and design, acquisition, analysis and interpretation of data, co‐drafted manuscript; BD: conception and design, interpretation of data, critical revision of manuscript; MH & GS: conception and design, analysis and interpretation of data, critical revision of manuscript; KT conception and design, analysis and interpretation of data, co‐drafted manuscript.

## FUNDING INFORMATION

Xin Rui Lim was in receipt of a Government of Ireland Postgraduate Scholarship (GOIPG/2019/2874) with additional support from the Research Office in Dundalk Institute of Technology.

## CONFLICT OF INTEREST

No conflicts of interest, financial or otherwise, are declared by the authors.

## Supporting information


Figure S1

## Data Availability

The data that support the findings of this study are available from the corresponding author, KDT, upon reasonable request.
