# Supplementary material for: Ca2+‐activated Cl− channels (TMEM16A) underlie spontaneous electrical activity in isolated mouse corpus cavernosum smooth muscle cells
Source: Physiol Rep. 2022 Nov 16;10(22):e15504. doi: 10.14814/phy2.15504 (PMC9669617; doi:10.14814/phy2.15504)
Supplement: Supplementary file 1 — Figure S1 [file PHY2-10-e15504-s001.pdf]

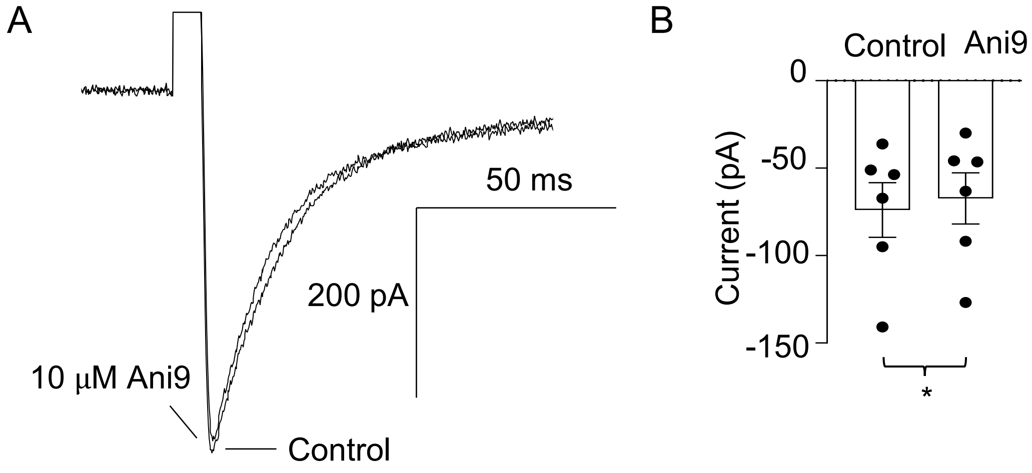

Figure S1: (A) Representative trace showing L-type Ca^{2+} current. *(B)* Summary data showing the effect of 10 µM Ani9 on L-type Ca^{2+} current. Although Ani9 caused a significant reduction in L-type Ca^{2+} current, this amounted to <10% (n = 6 cells from 6 animals; *$P < 0.009$*; paired t-test).
